# Snakebite envenomation in the Caribbean: The role of medical and scientific cooperation

**DOI:** 10.1371/journal.pntd.0006441

**Published:** 2018-07-12

**Authors:** Dabor Resiere, Hossein Mehdaoui, José María Gutiérrez

**Affiliations:** 1 Intensive Care Unit, University Hospital of Martinique, Fort-de-France, Martinique, France; 2 Instituto Clodomiro Picado, Facultad de Microbiología, Universidad de Costa Rica, San José, Costa Rica; Instituto Butantan, BRAZIL

Snakebite envenomation is a medical hazard that has a high public health impact, particularly in sub-Saharan Africa, Asia, and Latin America; other regions, such as Oceania and the Caribbean, are also affected by these envenomations [1]. Owing to its impact and to the fact that it predominantly affects impoverished rural populations, WHO adopted snakebite envenomation as a category A neglected tropical disease (NTD) in 2017, following a proposal presented by the government of Costa Rica and 17 additional countries [2]. WHO is currently working on a global plan to improve its control. Moreover, the WHO Executive Board has recently recommended a resolution on snakebite envenomation to the World Health Assembly [3]. These developments, together with additional efforts by diverse stakeholders, have prompted a growing interest on this public health problem in many regions. This viewpoint focuses on snakebites in the Caribbean, a region that includes a number of countries having venomous snakes, where this neglected disease has not received the attention it deserves.

The Caribbean encompasses a large group of countries distributed over a wide geographical range, with a rich historical, cultural, and natural heritage, and comprises a population of 50 million people. Venomous snakes live in Aruba, Belize, Guyana, French Guyana, Martinique, Suriname, Saint Lucia, and Trinidad and Tobago (Table 1). However, the epidemiology of snakebites in the Caribbean is largely unknown, and no systematic regional register of this disease exists. Previous studies have documented various aspects of snakebite envenomings in this region (see for example [4]). Most snakebite accidents in the Caribbean are inflicted by species of the family Viperidae (pit vipers), such as the fer-de-lance *Bothrops lanceolatus*, endemic to Martinique, and *B*. *atrox*, abundant in the Guyanas and Trinidad and Tobago (Table 1); fewer cases are inflicted by viperid species of the genera *Crotalus* and *Lachesis*, as well as by coral snake species of the family Elapidae (*Micrurus* spp.). As in other regions of the world [1], snakebites in the Caribbean mainly affect agricultural workers.

**Table 1:** Venomous snakes distributed in Caribbean countries, together with the population, estimated number of snakebites per year, and availability of antivenoms.

Country	Population	Venomous snakes^a^	Number of bites per year^b^	Availability of antivenom^c^
Aruba	104,822	*Crotalus durissus*	N/A^d^	N/A
Belize	387,879	*Agkistrodon bilineatus*, *Bothriechis schlegelii*, *Bothrops asper*, *Crotalus simus*, *C*. *tzabcan*, *Porthidium nasutum*	N/A	Polival-ICP (Instituto Clodomiro Picado)
French Guyana	287,351	*Bothrops atrox*, *B*. *bilineatus*, *B*. *brazili*, *B*. *taeniata*, *Crotalus durissus*, *Lachesis muta*, *Micrurus lemniscatus*, *M*. *surinamensis*, *Micrurus spp*.	50	Antivipmyn-Tri (Instituto Bioclon, Mexico)
Guyana	773,303	*Bothrops atrox*, *B*. *bilineatus*, *B*. *brazili*, *B*. *taeniata*, *Crotalus durissus*, *Lachesis muta*, *Micrurus lemniscatus*, *M*. *surinamensis*, *Micrurus spp*.	200	N/A
Martinique	400,000	*Bothrops lanceolatus*	30	Bothrofav (Sanofi Pasteur)
Saint Lucia	178,015	*Bothrops caribbaeus*	20	Polival-ICP (Instituto Clodomiro Picado)
Suriname	558,368	*Bothrops atrox*, *B*. *bilineatus*, *B*. *brazili*, *B*. *taeniata*, *Crotalus durissus*, *Lachesis muta*, *Micrurus lemniscatus*, *M*. *surinamensis*, *Micrurus spp*.	N/A	Various sources of polyvalent antivenoms
Trinidad and Tobago	1,353,895	*Bothrops atrox*, *Lachesis muta*, *Micrurus spp*.	N/A	N/A

^a^ Species distribution is based on the WHO webpage on antivenoms (http://apps.who.int/bloodproducts/snakeantivenoms/database/).

^b^ The estimated number of snakebites is based on the data by [15], except in Martinique and Saint Lucia, in which cases data are from information provided by local health authorities.

^c^ Information on availability of antivenoms is based on personal communications by colleagues from these countries.

^d^ N/A: Information not available.

Envenomations by viperid species are characterized by local tissue alterations at the site of venom injection (edema, pain, blistering, hemorrhage, myonecrosis, and dermonecrosis) and by systemic manifestations associated with bleeding, coagulopathy, cardiovascular, and renal disturbances and, in the cases of *B*. *lanceolatus* (Martinique) and *B*. *caribbaeus* (Saint Lucia), by severe thrombotic events in various organs [1, 4]. Antivenom is the universally accepted treatment of snakebites, and the clinical management of envenoming is centred on the intravenous administration of antivenom [1]. Unfortunately, this treatment is not available in several countries of the Caribbean, where it is most needed (Table 1). This highlights a very serious situation, as lack of antivenom or delay in antivenom treatment is associated with high mortality [5].

NTDs exert a heavy burden in the Caribbean [6, 7], and efforts have been performed to develop national and regional strategies to confront this group of diseases in this region and in Latin America with the participation of local health authorities and the Pan American Health Organization (PAHO) [8], and research priorities have been proposed for improved understanding of these diseases in the region [9]. However, since snakebite envenomation was formally added to the list of NTDs by WHO just recently, it has not been incorporated into the regional programs to combat NTDs. It is expected that this will change in the near future, along with current efforts being performed by PAHO and ministries of health. Snakebite envenomation was mentioned in the 55th Directive Council of PAHO in 2016 when discussing the elimination of NTDs in the region [10]. In this context, snakebites have not been considered a priority issue in the discussions of Caribbean health problems, and people interested in this topic have worked in isolation. It is about time that this trend is reversed and that appropriate attention be given to this health issue in the region.

The efforts carried out by individuals, groups, or at the national level should be complemented by a philosophy of medical and scientific cooperation in the region as a whole, since common health problems demand concerted approaches to study them and to develop solutions to reduce their impact. Along this trend, several initiatives have been undertaken (see for example [11]). The recent spate of hurricanes in some of the Caribbean countries has underscored the importance of regional approaches to confront such events, together with many health issues, within a framework of cooperation.

A concerted regional strategy is necessary to effectively reduce the burden of snakebite envenomation in the Caribbean. Such a strategy should include, at least, the following issues, in accordance with a global integrated strategy proposed to confront these envenomations [12]:

(a) Develop scientific research on various aspects of the biology of venomous snakes and the biochemical and toxicological characterization of snake venoms. In this regard, establishing cooperative research programs with groups in various countries of Latin America is a priority.
(b) In collaboration with research groups in various countries of Latin America and the Caribbean, assess the preclinical efficacy of antivenoms available in the region and communicate these results to national health authorities.
(c) Perform community-based studies to gather information on the main epidemiological aspects of envenomations, including the cultural perceptions of the local population on snakes and snakebites.
(d) Design prevention programs tailored to the local cultural contexts and aimed at reducing the incidence of envenomations.
(e) Perform investigations on the main clinical features of envenomations and on the safety and efficacy of antivenoms distributed in the region.
(f) Establish renewed procurement programs at the ministries of health and in coordination with PAHO to ensure the purchase and effective distribution of effective antivenoms.
(g) Design and implement education campaigns to inform the people on how to proceed in the event of a snakebite.
(h) Develop training programs for the health staff in the diagnosis and management of these envenomations.
(i) Establish programs to follow up with people once they leave the hospital after an envenomation to determine the incidence of physical and psychological sequelae and to implement intervention programs to improve their quality of life.
(j) Create or consolidate poison control centers with expertise in the topic of snakebite envenomation, which could provide timely and accurate information to the health staff or the general population on the prevention and management of snakebite envenomation.

Ongoing medical cooperation shows significant advances in other regions, such as in the Brazilian Amazon [13]. Likewise, collaborative regional efforts have been carried out in Latin America in the preclinical assessment of antivenom efficacy [14], although much remains to be done. Regional heath authorities need to consider establishing a regional register for accurate epidemiological assessment on the incidence of acute poisoning, particularly snakebite envenomation. The Caribbean Community (CARICOM) and other regional organizations should collaborate to develop more educational and informational systems and help to create regional poison centres. Efforts should also be made to provide antivenom to all hospitals in the region. Significant advances have already been performed by some governments and also by PAHO in the acquisition of antivenom to the most needy countries in the region.

The experience gained from ongoing cooperation between Martinique and other Caribbean countries in recent years can be used as a lead to develop common, firm relationships in training through a program of seminars and workshops for health workers in order to teach them the basic aspects of snakebite envenomation. It cannot be overstated that collective scientific research and continuous medical education among Caribbean professionals are the key to success in revitalizing health systems in the region. An example is the workshop on epidemiology and management of snakebites in French Guyana held at Cayenne, French Guyana from September 15 to September 16, 2017, under the auspices of the French Regional Health Agency (ARS) and the PAHO, with the participation of specialists from several countries from the Caribbean and elsewhere.

In addition to promoting regional cooperation on snakebites from a public health standpoint, there are socioeconomic, ecological, and cultural dimensions that need to be considered. In parts of these countries, snakes are regarded as animals that should be killed on sight because of the perceived threat to human life. More recently, as a result of greater emphasis on heritage preservation, *Bothrops caribbaeus* has been afforded a degree of protection in Saint Lucia, a step in the right direction. Prohibition on indiscriminate killing will be difficult to achieve unless the population is convinced that there is a greater benefit in preservation. Likewise, cultural perceptions on snakes and envenomations should be documented and preserved as part of the cultural heritage in these countries.

The relevance of protecting snake populations should be emphasized as a source of novel knowledge on the biology of these species and on the pharmacological richness of their venoms. Therefore, the strengthening of the local scientific capacity in Caribbean countries to study venoms will contribute to the appreciation of this treasure of their natural heritage. In addition to providing scientific knowledge on the ecology of snakes and on the medical aspects of envenomation, such developments may eventually bring novel lead compounds for the development of new drugs or research tools.
